# 251. Clinical Features and Outcomes of Pneumothorax and Pneumomediastinum in COVID-19

**DOI:** 10.1093/ofid/ofac492.329

**Published:** 2022-12-15

**Authors:** Sangeeta Adusumilli, Ashish Bhargava, Rene Franco

**Affiliations:** Ascension St.John Hospital, St Clair shores, Michigan; Ascension St. John Hospital; Wayne State University School of Medicine, Grosse Pointe Woods, Michigan; Ascension St.John, detroit, Michigan

## Abstract

**Background:**

Pneumothorax (PTX) and pneumomediastinum (PM) have been reported among hospitalized patients with COVID-19. It can occur among patients breathing spontaneously or as a result of barotrauma from invasive positive-pressure ventilation or from medical procedures. We aim to study the clinical features and outcomes of pneumothorax and pneumomediastinum within 48 hours of hospitalization among COVID-19 patients.

**Methods:**

We conducted a multicenter retrospective study among the hospitalized adults with COVID-19 who had pneumothorax and pneumomediastinum within 48 hrs. of admission between November 2020 and December 2021. Cases were identified using ICD 10 codes. Electronic medical records were reviewed after Institutional Board approval.

**Results:**

We identified a total of 21 patients, 12 (57%) only had PTX, 6 (28%) only had PM, and 3(14%) had both. Mean age for the cohort was 57 yrs, 13 (62%) were females, and 14 (67%) were whites. Chronic lung and end-stage renal diseases were noted among 9 (43%) patients followed by obesity in 9 (43%) and diabetes in 4 (19%). A total of 12 (57%) patients have smoked tobacco. At the time of hospitalization, 12 (57%) patients had oxygen saturation ≤94% and 9 (43%) had ≤90%. PTX and PM on admission chest x-ray were noted in 12(57%) and 4 (19%) respectively. 3 (14%) developed them after intubating and/ or after BiPAP. Patients were treated with steroids (90%), remdesivir (62%), interleukin-6 inhibitors (24%), and convalescent plasma (9%). Chest tube was placed in 7 (33%) patients and thoravent in 1 (5%) patient. Complications were septic shock (14%) and deep venous thrombosis (10%). There were 4(19%) deaths.

**Conclusion:**

Spontaneous PTX can be a presenting sign for COVID-19. We noted higher complications and mortality among the COVID-19 patients with PTX and PM than reported in literature. Clinicians should be aware of this potential occurrence, requiring close monitoring and aggressive management. Larger studies can further validate the findings of our study.

**Disclosures:**

**All Authors**: No reported disclosures.

